# Intravenous immunoglobulin and rituximab versus placebo treatment of antibody-associated psychosis: study protocol of a randomised phase IIa double-blinded placebo-controlled trial (SINAPPS2)

**DOI:** 10.1186/s13063-019-3336-1

**Published:** 2019-06-07

**Authors:** Belinda Lennox, Ksenija Yeeles, Peter B. Jones, Michael Zandi, Eileen Joyce, Ly-Mee Yu, Giuliano Tomei, Rebecca Pollard, Sally-Anne Vincent, Mio Shimazaki, Iona Cairns, Francis Dowling, Thomas Kabir, Thomas R. E. Barnes, Anne Lingford Hughes, Akram A. Hosseini, Timothy Harrower, Camilla Buckley, Alasdair Coles

**Affiliations:** 1 Department of Psychiatry, University of Oxford and Oxford Health NHS Foundation Trust, Warneford Hospital, Warneford Lane, Oxford, OX3 7JX UK; 2 School of Clinical Medicine and Department of Psychiatry, University of Cambridge, Box 189, Addenbrooke’s Hospital, Cambridge, CB2 2QQ UK; 3 Department of Neuromuscular Diseases, University College London Queen Square Institute of Neurology, and National Hospital for Neurology and Neurosurgery, Queen Square, London, WC1N 3BG UK; 4 University College London Institute of Neurology, The National Hospital for Neurology and Neurosurgery, Box 19, Queen Square, London, WC1N 3BG UK; 5 Primary Care Clinical Trials Unit, Nuffield Department of Primary Care Health Sciences, University of Oxford, Radcliffe Observatory Quarter, Woodstock Road, Oxford, OX2 6GG UK; 6 Research and Development, Devon Partnerships NHS Foundation Trust, Wonford House, Dryden Road, Exeter, EX2 5AF UK; 7 Cambridge Clinical Trials Unit, Cambridge University Hospitals NHS Foundation Trust, Addenbrooke’s Hospital, Coton House Level 6, Flat 61, Box 40, Hills Road, Cambridge, CB2 0QQ UK; 8 The McPin Foundation, 7–14 Great Dover Street, London, SE1 4YR UK; 9 Department of Medicine, The Centre for Psychiatry, Imperial College London, 7th Floor Commonwealth Building, Du Cane Road, London, W12 0NN UK; 10 Centre for Psychiatry, Imperial College London, Burlington Danes, Hammersmith Campus Imperial College, London, UK; 11 Department of Neurology, Queen’s Medical Centre, Nottingham University Hospitals NHS Trust, Derby Road, Nottingham, NG7 2UH UK; 12 Royal Devon and Exeter Hospital, Royal Devon and Exeter NHS Foundation Trust, Wonford, Barrack Road, Exeter, EX2 5DW UK; 13 Nuffield Department of Clinical Neurosciences, Oxford University Hospitals NHS Foundation Trust, John Radcliffe Hospital, West Wing, Headley Way, Oxford, OX3 9DU UK; 14 Department of Clinical Neurosciences, University of Cambridge, Box 165, Addenbrooke’s Hospital, Cambridge, CB2 2QQ UK

## Abstract

**Background:**

Evidence is conflicting about a causal role of inflammation in psychosis and, specifically, regarding antibodies binding to neuronal membrane targets, especially N-methyl-D-aspartate receptors. NMDAR, LGI1 and GABA-A antibodies were found more prevalent in people with psychosis than in healthy controls. We aim to test whether these antibodies are pathogenic and may cause isolated psychosis. The SINAPPS2 phase IIa double-blinded randomised controlled trial will test the efficacy and safety of immunoglobulin and rituximab treatment versus placebo for patients with acute psychosis symptoms as added to psychiatric standard of care.

**Methods:**

We will screen approximately 2500 adult patients with acute psychosis to identify 160 with antibody-positive psychosis without co-existing neurological disease and recruit about 80 eligible participants to the trial in the period from September 2017 to September 2021 across the UK. Eligible patients will be randomised 1:1 either to intravenous immunoglobulin (IVIG) followed by rituximab or to placebo infusions of 1% albumin followed by 0.9% sodium chloride, respectively. To detect a time-to-symptomatic-recovery hazard ratio of 0.322 with a power of 80%, 56 participants are needed to complete the trial, allowing for up to 12 participants to drop out of each group.

Eligible patients will be randomised and assessed at baseline within 4 weeks of their eligibility confirmation. The treatment will start with IVIG or 1% albumin placebo infusions over 2–4 consecutive days no later than 7 days from baseline. It will continue 4–5 weeks later with a rituximab or sodium chloride placebo infusion and will end 2–3 weeks after this with another rituximab or placebo infusion. The primary outcome is the time to symptomatic recovery defined as symptomatic remission sustained for at least 6 months on the following Positive and Negative Syndrome Scale items: P1, P2, P3, N1, N4, N6, G5 and G9. Participants will be followed for 12 months from the first day of treatment or, where sustained remission begins after the first 6 months, for an additional minimum of 6 months to assess later response.

**Discussion:**

The SINAPPS2 trial aims to test whether immunotherapy is efficacious and safe in psychosis associated with anti-neuronal membrane antibodies.

**Trial registration:**

ISRCTN, 11177045. Registered on 2 May 2017.

EudraCT, 2016-000118-31. Registered on 22 November 2016. ClinicalTrials.gov, NCT03194815. Registered on 21 June 2017.

**Electronic supplementary material:**

The online version of this article (10.1186/s13063-019-3336-1) contains supplementary material, which is available to authorized users.

## Background

It is broadly accepted that psychosis and schizophrenia are caused by genetic and environmental factors, associated with excess mesolimbic and mesocortical dopamine and the *N*-methyl-d-aspartate receptor (NMDAR) hypofunction [1]. However, a range of cell surface protein and immune genetic loci are associated with schizophrenia [1, 2] and there is increasing evidence for a role of inflammation in psychosis and schizophrenia [3, 4]. Antibodies binding to neuronal or glial membrane targets, especially NMDA receptors, in the brain were first described in 2007 causing encephalopathy, with psychiatric features in over two thirds that responded to immunotherapy [5]. In such patients, isolated psychotic episodes occur in 23/571 (4%), either at presentation (5/571) or at relapse (18/571) [6].

Open-label experience from several hundred patients suggests that immunotherapy is effective in the full-blown syndrome of encephalopathy associated with anti-NMDAR antibodies [7]. Antibodies to the NMDAR or the voltage-gated potassium channel complex (VGKC) were found in 3 (7%) out of 46 patients with first-episode psychosis, without other signs of encephalitis [8]. In a prevalence study (PPiP1) we replicated previous findings as 20 (8.8%) of 228 study participants with first-episode psychosis possessed these antibodies, with a rate of 4% in healthy controls [9]. The presence of NMDAR, of leucine-rich, glioma inactivated 1 (LGI1) and of gamma-aminobutyric acid type A (GABA-A) receptor antibodies in patients with a first episode of psychosis was found to be greater then in healthy controls. We found no cases of α-amino-3-hydroxy-5- methyl-4-isoxazolepropionic acid receptor antibodies (AMPAR), a greater rate of contactin-associated protein 2 (CASPR2) antibodies in controls than patients with psychosis, and no difference in VGKC antibodies between groups [9]. The VGKC antibody radioimmunoassay is no longer recommended clinically due to non-specificity [10]. Others have found NMDAR and LGI1 antibodies in cases and controls at a low rate [11]. The antibodies were, however, found in neurological and healthy controls (e.g. 13 of 56 NMDAR antibody-positive patients in a Cambridge and University College London (UCL) study had antibodies thought to be clinically irrelevant) [12].

There is considerable evidence that anti-NMDAR antibodies are usually pathogenic, down-regulating NMDAR currents both in vitro and in vivo [13–15]. The hypothesis underlying the SINAPPS2 trial is that these antibodies are pathogenic and may be responsible for isolated psychosis.

We have recently reported on 18 patients with NMDAR antibodies, nine of whom self-resolved or responded to antipsychotics. The remaining nine patients were resistant to up to three antipsychotics, so we treated them with corticosteroids combined with either plasma exchange (PLEX) or intravenous immunoglobulin (IVIG) to good effect, resulting in reduced serum antibody level, symptomatic remission and lowered disability in the majority of patients treated, with maintenance therapy for a few patients (mycophenolate or rituximab) [16].

We conducted an uncontrolled feasibility study (SINAPPS1) to test the referral pathway and acceptability of IVIG and PLEX with corticosteroids [17]. Nine (90%) out of 10 recruited participants with acute psychosis and the presence of anti-neuronal membrane antibody (antibody-associated psychosis) received immunotherapy within 14 days from the decision to treat at two acute hospitals in England. Four participants received IVIG and six were given plasmapheresis (combined with steroids in four patients). All participants were taking antipsychotic medication as prescribed by their psychiatrists. No adverse events were reported during the treatment. The severity of psychosis symptoms and the functioning of all participants improved within two months of the last treatment session. Symptomatic improvement was remarkable in participants with NMDAR antibodies and moderate in participants with VGKC or GABA-A antibodies. The SINAPPS1 trial has confirmed the feasibility of rapid and safe provision of immunotherapy in patients with psychosis in acute hospital settings. These findings informed the design of the phase IIa double-blinded randomised clinical trial SINAPPS2. The SINAPPS2 trial is investigating IVIG and rituximab versus placebo treatment without corticosteroids.

The SINAPPS2 trial tests the hypothesis that immunotherapy is an effective treatment for antibody-associated psychosis, either the first episode of psychosis or relapse following previous remission. The trial aims to show superiority of immunotherapy with IVIG and rituximab over conventional psychiatric care alone including antipsychotic therapy in patients with acute psychosis and central nervous system (CNS) membrane antibodies.

The trial was registered at ClinicalTrials.gov on 21 June 2017 (NCT03194815; https://clinicaltrials.gov/ct2/show/NCT03194815?term=NCT03194815&rank=1).

### Trial objectives

The primary objective is to test the efficacy of immunotherapy (IVIG and rituximab) in patients with acute psychosis associated with anti-neuronal membrane antibodies. The secondary objective is to test the safety of immunotherapy (IVIG and rituximab) in these patients.

## Methods

### Design

The SINAPPS2 trial is a phase IIa, double-blinded, multicentre randomised placebo controlled trial with 12 (up to 18 if required) months of follow-up. It will be conducted in five to eight acute National Health Service (NHS) hospital trusts across the UK with suitable facilities for treatment of this population.

### Participants

#### Inclusion criteria

1. Age 18–70 years.
2. Acute psychosis symptoms for at least 2 weeks but not longer than 24 months in the current episode. Acute psychosis is defined as rating 4 or more for at least one of the following Positive and Negative Syndrome Scale (PANSS) [18] items: P1, delusions; P2, conceptual disorganisation; P3, hallucinatory behaviour; N1, blunted affect; N4, social withdrawal; N6, lack of spontaneity; G5, mannerisms/posturing; and G9, unusual thought content. This may be either a first episode or relapse after remission. Remission is defined as having mild or absent symptoms of psychosis for at least 6 months.
3. Serum or cerebrospinal fluid (CSF) anti-neuronal membrane antibodies at pathological levels (including NMDAR, LGI1 and others, as determined by the Trial Steering Committee).
4. Given informed consent by a patient or their legal representative.

#### Exclusion criteria

1. Duration of current episode of psychosis greater than 24 months.
2. Co-existing severe neurological disease, including tumour, hippocampal sclerosis with refractory epilepsy, probable dementia with evidence of atrophy on brain imaging, moderate or severe learning disability, or any evidence of a current acute encephalopathy (for instance coma, seizures).
3. Hepatitis B.
4. Hepatitis C.
5. Human immunodeficiency virus.
6. IgG level < 3 g/L.
7. Previous malignancy (unless agreed with Chief Investigator (CI)).
8. Pregnant, breast feeding or inadequate contraception if female.
9. Hypersensitivity or absolute contra-indication to any trial drug, murine proteins or excipients.
10. Live vaccine within last 3 months.
11. Previous treatment with rituximab in the past 12 months.
12. Severe infection.
13. Severe heart failure.
14. Any other medical illness or disability that, in the opinion of the investigator, would compromise effective trial participation.
15. Concurrent enrolment in another Clinical Trial of Investigational Medicinal Products (CTIMP).

### Treatment medication

The interventional treatments are intravenous immunoglobulin (IVIG) followed one month later by rituximab, given as two infusions a fortnight apart.

### Placebo

Patients receiving control treatment will receive matched placebo solutions for IVIG (human albumin solution 1%) and rituximab (NaCl 0.9%), with NaCl 0.9% being used in place of methylprednisolone pre-medication to maintain blinding.

### Route of administration and maximum dosage allowed

The overall duration of the treatment period for an individual participant will be approximately 50 days. The study medication will be used at standard clinical doses, and delivered as per local protocols. IVIG intravenous infusion of 2 g/kg (ideal body weight) will be given over 2–4 days as tolerated. Most participants are expected to receive the drug over two 2–6 h periods. Placebo (1% albumin solution) will be given over 2–4 days.

The IVIG dosage is weight determined (ideal body weight). Patients below 152.4 cm (5 ft) in height will be treated using their actual body weigh. IVIG dosing will be rounded down to the nearest 5 g regardless of the calculated dose; that is, if the calculated dose is 39.9 g, they will receive 35 g.

The two infusions of 1 g rituximab will be given 14 days apart, over 2–4 h as tolerated, with 100 mg methylprednisolone pre-medication to reduce infusion-associated adverse effects. The first infusion will commence at 50 mg/h; after the first 30 min, it may be escalated in 50 mg/h increments every 30 min, to a maximum of 400 mg/h. In the absence of serious previous infusion-related reactions, the second infusion can be infused at an initial rate of 100 mg/h and increased by 100 mg/h increments at 30-min intervals, to a maximum of 400 mg/h. The matching placebo solution (sodium chloride 0.9%) will be given in an equal volume and duration to that used for rituximab infusion. No dosage modifications are permitted.

The IVIG and matched placebo are provided by Biotest Ltd. Rituximab (or bio-similar), matching placebo and pre-medication will be supplied by local site pharmacies. Details on identity, supply, packaging, labelling, label design, prescriptions, dispensing and distribution of investigational medicinal products are described in the trial pharmacy manual.

### Concomitant therapy

All prescribed concomitant medications will be recorded monthly from screening to the end of study visit. Participants will continue with antipsychotic medications as standard of care, under the supervision of their local psychiatrist. Participants will not receive any other investigational medicinal products during the study period.

### Randomisation

An Internet-based system provided by Sealed Envelope will be used to randomise participants and allocate treatment to individual patients [19]. Eligible patients will be randomly assigned 1:1 to either the IVIG/rituximab group or the placebo group before the baseline assessment. Each group will contain approximately 40 participants. The randomisation will be performed using the minimisation technique of Pocock and Simon [20], using NMDAR (yes vs no), LGI1 (yes vs no), GABA-A (yes vs no), duration of psychotic illness (less than 12 weeks vs 12 weeks and longer) and current smoking status (currently smoking vs currently not smoking) as dichotomous minimisation factors.

Notification of treatment allocation will be forwarded to the central trial coordinator, local investigator and local pharmacy by email upon randomisation.

### Method of blinding

Trial participants and investigators will be blinded to treatment allocation. Site pharmacies and other authorised delegates as required will be unblinded. Treatment products/pre-medication and the corresponding control will have identical presentations in product appearance, packaging and labelling.

### Primary outcome

The primary outcome is the time to start of symptomatic recovery. Symptomatic recovery is defined as symptomatic remission sustained for at least 6 months [21]. Symptomatic remission is defined as a score of 3 or less on each of the following PANSS [18] items: P1, P2, P3, N1, N4, N6, G5 and G9.

### Secondary outcomes

1. Time to symptomatic remission

Symptomatic remission is defined as a score of 3 or less on each of the PANSS items P1, P2, P3, N1, N4, N6, G5 and G9, regardless of whether or not this response is sustained for 6 months (i.e. response, but does not reach the duration criterion for recovery).

2. Relapse

Relapse outcome is defined as the proportion of participants who relapse at least once during the 12-month follow-up period. Relapse is defined as a score of 4 or more on at least one of the PANSS items P1, P2, P3, N1, N4, N6, G5 or G9 after initial symptomatic remission occurred during the 12-month follow-up period.

3. Number of adverse events (AEs) over the 12-month follow-up period

The total number of AEs, total number of serious adverse events (SAEs) and total number of AEs with grade III or IV as reported in the AE CRF over the 12-month follow-up period will be presented by randomised group.

4. Number of serious infections

The total number of reported serious infections and the number of serious infections per participant over the 12-month follow-up period will be reported for each randomised group.

5. Proportion of patients reaching 20%, 30% and 40% reduction in PANSS total score over the 12-month follow-up period.

### Trial assessments

**Fig. 1:**
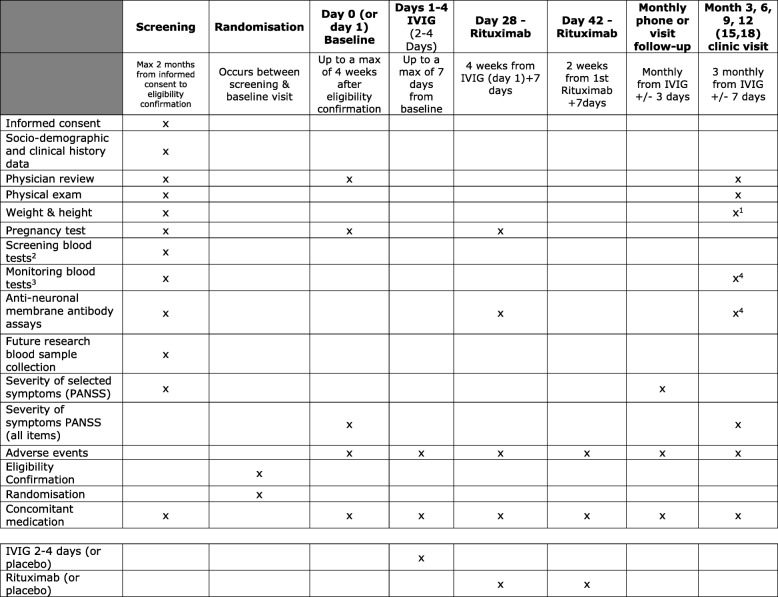
Trial assessments. ^1^Only weight at the end of study visit. ^2^The required screening blood tests are urea, electrolytes, calcium, HIV, hepatitis B, hepatitis C, syphilis, pneumococcal and varicella serology. ^3^Full blood count, liver function tests and immunoglobulins. ^4^Only at month 6, 12 and end of trial visit. IVIG intravenous immunoglobulin, PANSS Positive and Negative Syndrome Scale

Schedule of assessments and visits is summarised in Fig. 1 according to the SPIRIT statement [22, 23].

### Anti-neuronal membrane antibody assays

We use a live cell-based assay system [24] to identify serum anti-neuronal membrane antibodies. We have identified criteria to attempt to distinguish clinically irrelevant cases of positive anti-NMDAR and VGKC complex antibodies from pathogenic antibodies [12, 25].

Anti-neuronal membrane antibody assays will be performed for NMDAR, LGI1 and GABA-A antibodies. During the course of the study, other relevant anti-neuronal antibodies may be discovered and the protocol allows for these to be included, as determined by the Trial Steering Committee. Anti-neuronal membrane antibodies will be assessed at screening, the first rituximab treatment visit (28–35 days), at 6 and 12-month follow-up, and at the end of study clinical visit.

### Safety monitoring

The screening blood tests will be performed only at the screening visit and will include urea, electrolytes, calcium, HIV, hepatitis B, hepatitis C, syphilis, pneumococcal and varicella serology.

The monitoring blood samples will be taken at the screening visit, at 6 and 12-month follow-up visits and at the end of study clinical visit. Monitoring blood tests include FBC, liver function tests (LFT) and immunoglobulin levels.

Pregnancy is an exclusion factor. A pregnancy test (urine sample) will be conducted with female participants at the screening visit to confirm the eligibility of a potential trial participant. A further pregnancy test will be performed at the baseline visit (no longer than 7 days before the first dose of IVIG). Another and final pregnancy test should be taken no longer than 7 days before the first dose of rituximab.

The trial participants’ or their partners’ pregnancies during the trial period and up to 2 years after trial enrolment will be reported using the relevant Pregnancy Reporting Forms within 24 h of notification.

### Primary outcome measure

The Positive and Negative Syndrome Scale (PANSS) [18] is a clinician/researcher-rated 30-item scale for the assessment of the severity of symptoms of schizophrenia based on information obtained from a patient during the interview and from their family members and clinicians. Each item is rated from 1 (absent) to 7 (extreme) and they are grouped into three subscales: Positive Scale, P (7 items); Negative Scale, N (7 items); and General Psychopathology Scale, G (16 items). The sum of individual ratings within each subscale gives score ranges of 7–49 for the Positive Scale and Negative Scale and of 16–112 for the General Pathology Scale. A higher score indicates severe symptoms.

The primary outcome will be assessed by eight selected PANSS items [21]. These include three Positive Scale items (P1, delusions; P2, conceptual disorganisation; P3, hallucinatory behaviour), three Negative Scale items (N1, blunted affect; N4, passive/apathetic social withdrawal; N6, lack of spontaneity) and two items from the General Pathology Scale (G5, mannerisms/posturing; G9, unusual thought content).

PANSS assessments will be conducted by trained researchers. Their training includes PANSS training by certified trainers followed by observed role-play interviews with feedback, observed mock interviews with non-patients and patients with psychosis, and mock-interview videoing and analysis. Thereafter, there will be periodical refresher training with certified PANSS trainers and researchers’ independent rating of individual patient video interviews over the trial period.

### Trial procedures

#### Identification of potential participants and enrolment

**Fig. 2:**
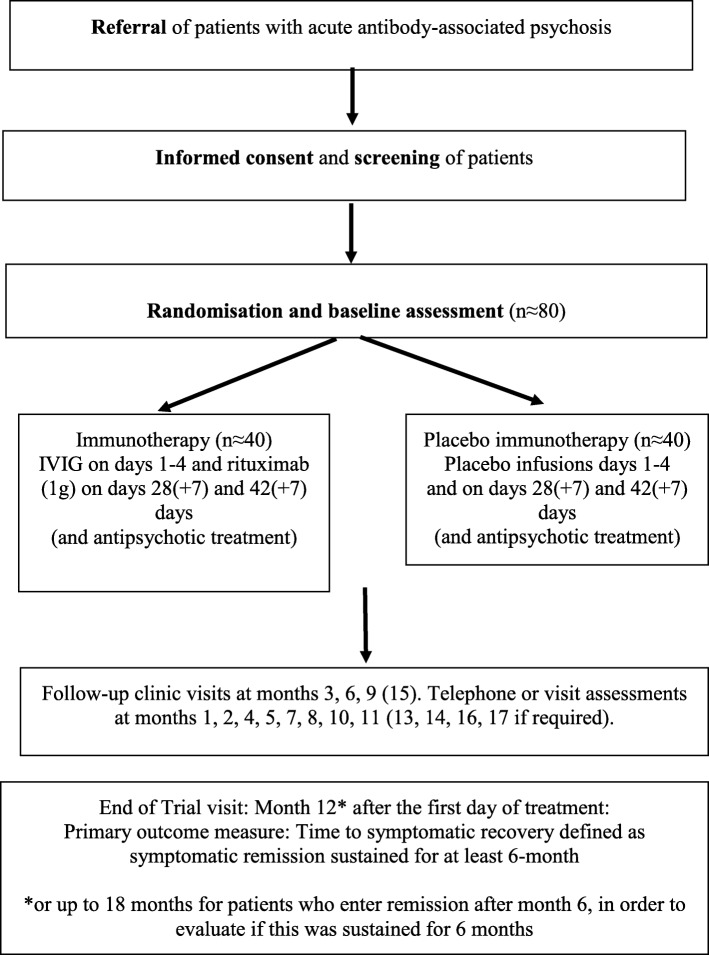
Trial flow chart. IVIG intravenous immunoglobulin

Participants will be identified to a SINAPPS2 trial neurologist either by referral from their mental health care team after receiving positive results for their routine antibody testing in clinical practice or after having been tested within the screening study on the prevalence of antibody-associated psychosis (PPiP2 study, REC ref: 12/EE/0307). The trial flow is summarised in Fig. 2.

Antibody-positive patients will be given the patient information sheet and members of the research team will clarify any information which may preclude recruitment. Informed consent will be obtained by a researcher or a doctor at the screening visit before trial-specific assessments.

Participants lacking the capacity to make a decision to take part in the study will be approached to be enrolled in the study under the conditions and principles listed in the Medicines for Human Use (Clinical Trials) Regulations [26].

A Personal Legal Representative (PeLR) close to that incapacitated patient, aware of his/her wishes and independent of the research, should be approached first to provide written consent. If it is not possible to approach a personal legal representative or there is no one sufficiently close to the potential participant who is willing or able to take on the role, then an independent clinician will be nominated to act as Professional Legal Representative (PrLR) to fulfil this role. The PrLR in the SINAPPS2 trial is defined as a person who is not connected with the conduct of the trial, specifically not the Sponsor of the trial, nor a person employed or engaged by acting under the arrangements with the Sponsor, and who undertakes activities connected with the management of the trial, not an investigator of the trial and not a health care professional who is a member of the investigators’ team for the purposes of the trial.

Participants who regain capacity will be informed of the clinical trial and consent to continue will be sought at that time as per the usual consenting process.

If a participant consents to the trial but later becomes incapacitated, the original consent given remains valid after the loss of capacity, providing that the trial has not significantly altered.

### Screening, baseline, treatment and follow-up

#### Outline

Screening assessments and procedures, summarised in Fig. 1, will only be conducted after a participant has signed the consent form. Participants will be randomised after confirmation of their eligibility, and before the baseline visit. The eligibility must be confirmed within a maximum of 2 months from the informed consent. There is no other time limit for randomisation. The baseline visit will be scheduled a maximum of 4 weeks after confirmation of eligibility and a maximum of 7 days before IVIG/placebo treatment. Rituximab first infusion will follow after 4–5 weeks and rituximab second infusion 2–3 weeks later. All trial participants will be required to attend follow-up clinical visits at 3, 6, 9 and 12 months, which will be an end of study visit. Participants may need to be followed for longer and attend a clinical visit at month 15 and 18 if the primary outcome data need to be obtained beyond month 12. In addition, a monthly follow-up brief telephone or visit assessment will be administered by researchers. Anonymised data will be transferred in Case Report Forms (CRFs) by the trial researchers and clinicians. All CRFs will be completed in the manner, and within the timelines, detailed in the CRF-completion guidelines and sent to the trial coordinator at the Cambridge Clinical Trials Unit (CCTU). The CRFs will be accessible to trial coordinators, data managers, the investigators, clinical trial monitors, auditors and inspectors as required. All trial data will be entered and stored in a MACRO database. A trial-specific data management plan will describe in detail the data management processes.

1. Screening visit and Assessments (Visit 1)

The screening visit and assessments will usually be performed at the SINAPPS2 site local clinical research facility, but may also be performed at outpatient clinics. Informed signed consent will be obtained and this will be followed by screening assessments to establish eligibility. These will include physician review, physical examination, height and weight assessments, pregnancy test (urine sample) if appropriate, collection of demographic data and disease history, screening blood tests, monitoring blood tests, anti-neuronal membrane antibody assays, blood sample for future research, assessment of PANSS items P1, P2, P3, N1, N4, N6, G5 and G9, and concomitant medications.

2. Baseline visit and assessments (Visit 2 (day 0, maximum 7 days before IVIG/placebo treatment))

The baseline visit will follow confirmation of a participant’s eligibility and randomisation. It will be arranged a maximum of 4 weeks after confirmation of eligibility. Participants will be reviewed by a physician for the period between screening and baseline dates. Clinical assessment (PANSS), assessment of adverse events and concomitant medication will be administered by a researcher.

3. IVIG or placebo treatment (Visit 3 (days 1–4))

The first treatment will follow the baseline visit (0–7 days after baseline) and will be given in an appropriate outpatient or inpatient setting, as determined by local practice and clinical need. Data on adverse events and concomitant medication will be obtained.

4. Rituximab or placebo 1 treatment (Visit 4 (day 28, + 7 days)

Rituximab or placebo first treatment will be given in an appropriate setting, as determined by local practice and clinical need. Anti-neuronal membrane antibody assays and a pregnancy test (a maximum of 7 days before the first dose of rituximab/placebo) will be taken and data on adverse events and concomitant medications will be recorded.

5. Rituximab or placebo 2 treatment (Visit 5 (day 42, + 7 days)

Rituximab or placebo second treatment will take place 14 (+ 7) days after the rituximab first treatment and data on adverse events and concomitant medications will be collected.

6. Follow-up clinical visits (months 3, 6 and 9 (12 and 15), ± 7 days)

Each study participant will be followed for 12 months from the day of first treatment (IVIG/placebo). There will be an additional maximum 6 months of follow-up, if required, to assess the primary outcome if the participant enters clinical remission between months 7 and 12 (to include follow-up for a further 6 months from the point of entering clinical remission). Participants will be assessed every 3 months (at months 3, 6 and 9 (and months 12 and 15 if required for participants requiring longer follow-up) ± 7 days). At follow-up clinical visits there will be a physician review, a physical examination, assessment of severity of symptoms (PANSS), adverse events and concomitant medications. In addition, monitoring blood tests and anti-neuronal membrane antibody assays will be performed at month 6 (and month 12).

7. End of trial visit (month 12 (or 15 or 18) ± 7 days).

Participation will end 12 months after the first day of treatment, or at 15 or 18 months if required. Assessments will include a physician review, physical examination, weight, monitoring blood tests, anti-neuronal membrane antibody assays, assessment of PANSS, adverse events and concomitant medications.

Participants will return to a normal standard of care after participation in the trial, which is likely to be a local psychiatry review, with a neurologist’s input and review if antibodies and symptoms return.

8. Monthly telephone or visit follow-up assessments will be conducted by researchers to assess the primary outcome, adverse events and concomitant medication at months 1, 2, 4, 5, 7, 8, 10 and 11 (months 13, 14, 16 and 17 if required) ± 3 days. A month-one follow-up assessment will be conducted at the first rituximab treatment visit on day 28 (+ 7).

### Emergency unblinding

In the event of a clinical adverse event, where knowledge of the treatment allocation will alter management, the treating physician may unblind the patient using the web-based randomisation system (through a procedure documented in the trial manual/randomisation system manual). It is the responsibility of the treating physician to promptly document and explain any unblinding to the Sponsor.

### Withdrawal

Patients who have withdrawn from the allocated treatment will remain in the trial and be followed up as set out in the protocol, unless they withdraw their consent for this trial. Any data and samples collected up until that point of consent withdrawal will continue to be used in the trial. However, no further information will be collected or tests performed. Participants who withdraw between randomisation and treatment will be replaced.

In addition, participants must be withdrawn from their allocated treatment arm if they develop severe encephalopathy (e.g. typical of NMDAR encephalitis or LGI1-antibody encephalopathy), which would necessitate withdrawal to facilitate best standard of care of management for these conditions [7]; the participant develops severe reactions to the Investigational Medicinal Products (IMP) (e.g. anaphylaxis to first rituximab infusion, aseptic meningitis to first dose of IVIG); or a female participant becomes pregnant or will no longer use contraception.

### Trial restrictions

Women of childbearing potential are required to use adequate contraception for the duration of the trial and for 12 months after the last treatment. Men are required to use adequate contraception for the entire duration of the trial and for 90 days after the completion of the last treatment. Male participants should refrain from donating sperm for the duration of the trial and for 12 months thereafter.

### Sample size

To estimate the control rate for the primary outcome efficacy measure, we have re-analysed the raw data of the National EDEN study [27]. This study consisted of 1000 patients with psychosis (antibody associations not known); 19% of those, who were symptomatic at entry, experienced sustained remission as per the Andreasen et al. criteria [21] on standard antipsychotic therapy at 1 year. The 50% remission rate on immunotherapy that we have selected is a conservative estimate; in anti-NMDAR encephalopathy, remission may be as high as 85% at 1 year following IVIG and rituximab [7]. We agreed on 50% as a clinically meaningful and important remission rate that from a patient perspective would make participation in the trial acceptable. The Trial Steering Committee (TSC) and statistician will analyse recruitment and dropout rates at months 12 and 24 of the trial, and consider altering the recruitment strategy. We have chosen a sample size to target a time-to-remission hazard ratio of 0.322 with a one-sided type I error rate of 5% and a power of 80%, standard for a stage II clinical trial. This target hazard ratio comes from assuming a 1-year remission probability of 0.2 in the control arm and 0.5 in the experimental arm and assuming the time-to-remission distribution is exponential with proportional hazards. With all participants followed up for 1 year, a sample size of 56 participants equally randomised between the control and experimental arms is required. The sample size was calculated using NCSS PASS v14. We intend to recruit about 80 participants, allowing dropout of 12 participants per arm. This level of dropout is a cautious estimate because, although a high dropout is often seen in drug trials in psychosis, we are not requiring continued compliance with medication regimes after the infusions of IVIG and rituximab.

### Statistical analysis methods

The final analysis will use a Cox proportional hazards model with fixed effects for minimisation covariates. The primary analysis will be per protocol rather than intention to treat, although both will be done. Secondary and exploratory outcomes will be analysed with Cox models (time to any remission), logistic regression models (relapse rate, proportion of participants with serious adverse events) and Poisson models (number of adverse events). The per-protocol population is defined as all participants who have received all protocol-defined IMPs, and have no major protocol violation as defined prior to analysis. A full detailed analysis plan will be prepared by a statistician who is independent from the study.

Data on trial progress such as recruitment and participant dropout at months 12, 24 and 36 of the trial (after activation of the first site) and quality of data will be reported to both the Trial Steering Committee and the Data Monitoring Committee (DMC). The Trial Steering Committee will consider opening additional sites and/or modifying eligibility criteria if recruitment rates are below those expected. There will be no interim analyses of efficacy.

### Trial management

The ethical and safety aspects of the trial (e.g. rate of severe adverse events/severe adverse reactions) will be reviewed by the Data Monitoring Committee.

The DMC will meet (virtually if necessary) at least annually to consider the safety data emerging from the trial. The committee may request unblinded data and more frequent listings if necessary. The DMC reports to the CI and the Trial Steering Committee.

The TSC will provide overall supervision with respect to the conduct of the trial. Full details of the TSC and DMC membership and remit can be found in the TSC and DMC Charters. Both are available from the CI on request.

The Trial Management Group (TMG) (CI, Principal Investigators (PIs), trial statistician, trial coordinator, psychiatrists and project manager) meets weekly, manages the trial overall and discusses key decisions.

### Data protection and patient confidentiality

All investigators and trial site staff involved in this trial will comply with the requirements of the Data Protection Act 1998 and Trust Policy with regards to the collection, storage, processing and disclosure of personal information and will uphold the Act’s core principles.

Only anonymised data will be received by the trial coordination team. Patient personal data will be kept locally at each site in secure locked cabinets or electronically on encrypted secure drives.

### Sponsor

The trial Sponsor is Cambridge University Hospitals NHS Foundation Trust & the University of Cambridge (Mrs Carrie Bayliss, Cambridge Clinical Trials Unit, Coton House Level 6, Box 401, Addenbrooke’s Hospital, Hills Road, Cambridge CB2 0QQ, UK; cctu@addenbrookes.nhs.uk). The trial Sponsor did not have a role in the study design, collection, management, writing of the paper and decision to submit the paper for publication, and it has no ultimate authority over any of these activities.

### Insurance

Cambridge University Hospitals NHS Foundation Trust, as a member of the NHS Clinical Negligence Scheme for Trusts, will accept full financial liability for harm caused to participants in the clinical trial through the negligence of its employees and honorary contract holders. There are no specific arrangements for compensation should a participant be harmed through participation in the trial if no-one has acted negligently.

The University of Cambridge will arrange insurance for negligent harm caused as a result of protocol design and for non-negligent harm arising through participation in the clinical trial.

### Monitoring, audit and inspection

All trial documentation and related records will be available should a Medicines and Healthcare products Regulatory Agency (MHRA) Inspection or a monitoring visit or audit of the Sponsor’s representative occur. The Sponsor’s monitoring frequency will be determined by an initial risk assessment performed prior to the start of the trial. A detailed monitoring plan will be generated detailing the frequency and scope of the monitoring for the trial. Throughout the course of the trial, the risk assessment will be reviewed and the monitoring frequency adjusted as necessary. Remote monitoring will be conducted for all participating sites. The scope and frequency of the monitoring will be determined by the risk assessment and detailed in the Monitoring Plan for the trial.

### Publications policy

Ownership of the data arising from this trial resides with the trial team. On completion of the trial, the data will be analysed and tabulated and a Final Trial Report prepared. The funders of the trial will be acknowledged. Participants will be informed of the outcome of the trial through the trial website. The trial findings will be reported in peer-reviewed journals.

## Discussion

The SINAPPS2 trial has significant potential implication for the understanding and management of psychosis. Consequently, it could have a profound impact on the improved health of a group of patients presenting with psychosis.

Anti-inflammatory treatments have been shown to have some beneficial effects in schizophrenia. Although immunotherapy is already used to treat psychosis patients with neurological disease, this is the first randomised controlled trial designed to test immunotherapy for patients with anti-neuronal membrane antibodies and acute psychosis without co-occurring severe neurological disease. The SINAPPS2 trial will contribute to a limited but growing body of evidence about the role of pathogenic anti-neuronal membrane antibodies in psychosis.

The data from open non-randomised studies [16, 17] using IVIG and PLEX for patients with acute psychosis and presence of anti-neuronal membrane antibodies are promising. Findings suggest that IVIG and PLEX have a positive safety profile, and IVIG but not PLEX is acceptable to participants. Psychosis symptoms of participants in both studies were improved. The SINAPPS2 trial will investigate patients’ views on acceptability and safety of combined IVIG with rituximab. The clinical pathway for these patients is still not clear and may be a challenge during the trial, but the SINAPPS2 trial has the potential to explore and introduce an efficient clinical pathway for assessment and treatment of patients with antibody-associated psychosis in neurology clinics.

## Limitations

The study will provide evidence on the efficacy of treatment but its effectiveness would still have to be tested in a future phase of the trial. All participants will continue with their antipsychotic medication treatment during the trial. This may mask the detection of the actual effect of immunotherapy or distinguishing effects of each treatment (antipsychotics and immunotherapy). The design of the trial, specifically the method of the IMP administration, has introduced potential selection bias. The trial participants are required to endure intravenous treatments over several hours per day. This may be difficult for patients with extremely severe psychosis symptoms and they may not be initially included in the trial. However, they may be re-approached when the severity of their symptoms no longer prevents them undertaking lengthy intravenous treatment.

## Trial status

Valid trial protocol at time of submission: v3; 20/12/2018.

Participants are currently being recruited and enrolled. The recruitment began on 25 September 2017 and the first participant was enrolled on 27 September 2017. At the time of submission there were seven enrolled participants. The estimated end date of the recruitment is 31 December 2020 and the trial completion date is 31 December 2021. The populated SPIRIT [22, 23] checklist is provided as Additional file 1.

Contact: www.sinapps.org.uk; sinapps@psych.ox.ac.uk; @SINAPPS_group; @SINAPPSgroup.

## Additional file

Additional file 1: SPIRIT 2013 checklist: recommended items to address in a clinical trial protocol and related documents. (DOC 120 kb)

## Abbreviations

AE: Adverse event
CCTU: Cambridge Clinical Trials Unit
CI: Chief Investigator
CRF: Case Report Form
CSF: Cerebrospinal fluid
CTIMP: Clinical Trial of Investigational Medicinal Products
DMC: Data Monitoring Committee
FBC: Full blood count
GABA-A: Gamma-aminobutyric acid subunit A
GCP: Good Clinical Practice
HIV: Human immunodeficiency virus
IMP: Investigational Medicinal Product
IVIG: Intravenous immunoglobulin
LFT: Liver function tests
LGI1: Leucine-rich glioma inactivated 1 antigen
MHRA: Medicines and Healthcare products Regulatory Agency
NMDAR: N-methyl-D-aspartate receptor
PANSS: Positive and Negative Syndrome Scale
PeLR: Personal Legal Representative
PLEX: Plasma exchange
PrLR: Professional Legal Representative
REC: Research Ethics Committee
SAE: Serious adverse event
TMG: Trial Management Group
TSC: Trial Steering Committee
VGKC: Voltage-gated potassium channel complex

## Authors’ information

MZ is supported by the National Institute for Health Research University College London Hospitals Biomedical Research Centre.
